# Copper/Zinc-Superoxide Dismutase in Human Epidermis: An Immunochemical Study

**DOI:** 10.3389/fmed.2019.00258

**Published:** 2019-11-13

**Authors:** Giovanna G. Altobelli, Susan Van Noorden, Vincenzo Cimini

**Affiliations:** ^1^Department of Advanced Biomedical Sciences, Medical School, Federico II University of Naples, Naples, Italy; ^2^Department of Histopathology, Imperial College London, Hammersmith Hospital, London, United Kingdom

**Keywords:** Cu, Zn-superoxide dismutase, human skin, immunochemistry, skin tumors, densitometry

## Abstract

The localization of copper and zinc-superoxide dismutase in normal and neoplastic human skin was examined with immunochemical techniques. Skin samples were taken from males and females of different ages, UV exposed and non-exposed areas and basal-/spino-cellular carcinomas. The enzyme was localized diffusely in the cytoplasm and was also found in the nuclei of epidermal cells, endothelial cells and other dermis cell types. The dismutase content in the epidermis was higher in males than females, UV-exposed than non-exposed and young than old people. In the tumors, the enzyme content of the superficial epidermal layers was higher than in the deep tumoral epithelial cells. These data suggest that the localization of Cu, Zn-SOD in skin tissues reflects the gender and age of the subject, the cell types and their normal or diseased state. Further studies based on the investigation of systemic changes of this enzyme in physiological and pathological epidermis could provide additional information on tumor cell generation.

## Introduction

Cells produce energy through a process of cellular respiration, reducing molecular oxygen to water. During this process, small amounts of partially reactive oxygen forms are also generated as the inevitable by-product of mitochondrial respiration. These include superoxide anion, hydrogen peroxide, and hydroxyl radical. Cells are equipped with defense systems to prevent the damage caused by these products. The skin's antioxidant defense system is regulated by a closely interconnected network, in which a change in the reduced state or the concentration of a component compromises other components of the system (1). An imbalance between production and elimination of radicals produces oxidative stress, which is associated with cellular damage in many pathological conditions. An intact system is therefore essential to protect the skin from damage due to reactive oxygen species (ROS). Among the many enzyme and non-enzyme systems that contribute to the inactivation of free radical reactions, superoxide dismutase (SOD) is expressed in the skin cells. Three types have been identified in humans: copper and zinc-superoxide dismutase (Cu, Zn-SOD), Mn-SOD, and extracellular SOD. The different distribution of copper-zinc and manganese SOD seems to suggest their different function (2). Under normal conditions there is enough Cu, Zn-SOD and Mn-SOD in the cells to eliminate the intracellular superoxide anion (3). Recent studies have shown that Cu, Zn-SOD is not only contained in most tissues as well as skin but is also secreted by various cell types (4) and furthermore contained in the serum (5).

The skin is in direct contact with the external environment, which induces the production of ROS in aging, skin diseases and cancer. Aging due to sunlight, photo-aging, depends on the degree of exposure and the type of skin. The greatest histological changes of natural aging and photo-aging appear in the dermis but after exposure to UVA and UVB rays, there is also a strong depletion of antioxidant enzymes in the keratinocytes of the stratum corneum and fibroblasts with a consequent increase in the level of protein oxidation (6).

The skin is the organ most exposed to environmental insults, consequently it is the tissue generally most affected by tumors. In skin cancer due to chronic UV exposure there is a reduction in antioxidant defenses, which can lead to clonal expansion of “initiated” cells. Skin cancer constitutes about 30% of all cancers diagnosed in the world and solar UV radiation, particularly the UVB component, causes about 90% of skin cancer.

Histologically, the tumor cells resemble those of the basal layer of the epidermis. There are two characteristic aspects: multifocal growth, in which the neoplastic cells originate from the epidermis and extend laterally, and nodular lesions, in which the cells grow deep down in the dermis immersed in a mucinous matrix, and often surrounded by many fibroblasts and lymphocytes.

Most of the data cited on the distribution of SOD in the skin refer to mammalian, non-human skin. Several studies demonstrate the presence of SOD in human skin but most of them have been conducted on the whole skin. There are, however, a few that have evaluated the characteristics of this enzyme, separating the epidermis from the dermis. As we have already pointed out, the epidermis and dermis have different cell populations, vascularity and intercellular constituents. In addition, the epidermis is more exposed to external agents, so the production of ROS and the defense systems against them are probably different (7). Moreover, most of this work has been carried out on cell cultures of animal models and consists mainly of biochemical studies, which do not allow a precise evaluation of the enzyme localization. Therefore, the aim of this study is to confirm, through morpho-functional studies, the presence of Cu, Zn-SOD in the human epidermis and study its distribution throughout its thickness and in various areas of the body surface, comparing the expression of this enzyme in the epidermis of healthy subjects and of patients with skin tumors, in particular spinocellular and basal cell carcinomas.

## Materials and Methods

Skin samples were taken from the departmental archive. Some samples, taken during surgery, had been immediately frozen and stored at −80°C for immunochemical analysis, while most were formalin-fixed and paraffin-embedded. The health status of each patient, together with age, sex, and area from which the sample had been taken, were recorded. At least three specimen of the same sample in each group of different areas, genders and other conditions were studied. As this is a retrospective study that does not involve clinical experimentation and uses already available biological material, for which formal consent may not have been needed, specific ethical approval is not required.

### Immunohistochemistry

The dilutions of the antibodies used are shown in Table S1. The antibodies were validated for specificity by the manufacturers. Mouse monoclonal anti-SOD was used to detect human skin Cu, Zn-SOD. Rabbit anti-ACTH was used on rat pituitary sections and mouse anti-insulin on hamster pancreas sections as positive method controls. After incubation overnight at 4°C, the primary antibody was removed by washing in PBS. The secondary antibody was applied for 1 h at room temperature. Alkaline phosphatase was visualized with naphthol phosphate and Fast Red. Peroxidase was developed with H_2_O_2_ and DAB. Nuclei were counterstained with Mayer's hematoxylin. Peroxidase and alkaline phosphatase were variably used to detect Cu, Zn-SOD, but for densitometry, after peroxidase development, nuclei were stained with Basic Methyl Green for 8 min at 60°C; then differentiated through quick washes in distilled H_2_O. Methyl Green was chosen because it lends itself well to quantitative histophotometric determinations (8); as it does not absorb at the wavelength of 480 nm which is used for DAB (9), it does not interfere with the signal expressed by SOD during densitometric measurements.

### Negative Controls

As a negative control, the primary antibody was replaced by the normal serum of the species providing the primary antibody, generally at the same dilution as the antibody, or PBS.

### Densitometric Analysis

Four μm-thick, formalin fixed-paraffin embedded sections treated with the antigen-specific antibody were observed with a Leica DMLB light microscope equipped with a Sony XC-77 CE camera. The images were digitized and processed for densitometric analysis according to the Image J program (imageJ.nih.gov). Briefly, the immunoreactive structures, labeled by the precipitated DAB during the development of the reaction, absorbed monochromatic light filtered at 480 nm wavelength (metric sized interference filters, Edmund Scientific Company, USA). The amount of light passing through the immunostained structures in the section, was measured. Optic calibration was performed to optimize measurements. The data were represented as mean ± SD.

### Statistical Analysis

Measurements were made at the level of the various layers of the epidermis for healthy subjects, while for basal- and spinocellular carcinomas the absorbance at the level of the surface epidermis and in the tumor areas invaginated in the dermis was measured. At least three slides were used for each condition. Ten fields per section were randomly selected, and six to ten optical density (OD) measurements of the immunoreactive structures per unit area were made in each field. At each measurement, the system averages the measured values in the area of interest (AOI) and provides a unique value, representative of the average of all the values expressed in that specific area. The densitometric value of each section was obtained from the average of the mean values of the ten selected fields. This measurement was performed three times on different sections of the same sample. *T*-test statistical analysis was performed only where necessary. In that analysis, a *p* < 0.05 was considered statistically significant.

### Western Blot

Unfixed skin (frozen samples) was homogenized on ice for 30 min in Tris-buffered saline, pH 7.5 (TBS) containing 0.05 M Tris-HCl, 50 mM NaCl, 1% NP-40, 2 mM EDTA, 2 mM PMSF, 5 μg/mL leupeptin, 5 μg/mL pepstatin. A sample of rat cerebellum was used as a positive control. An equal amount of protein from each sample was loaded, resolved by SDS-PAGE and transferred subsequently to a nitrocellulose membrane, Immobilon (Millipore Corporation, Bedford, MA), that then was incubated overnight at 4°C with rabbit anti-SOD1. After 3 washes in TBS containing 0.1% Tween 20 (TBS-Tween), the secondary antibody was added, an anti-rabbit immunoglobulin conjugated with horseradish peroxidase (Amersham Pharmacia Biotech UK Limited), diluted 1/2000 in TBS-Tween for 1 h at RT. Finally, protein bands were detected by an enhanced chemiluminescence method (Amersham Bio-sciences). The solution used to detect peroxidase consisted of hydrogen peroxide (peroxidase substrate), luminol (a luminescent substance), and phenol. The peroxidase oxidizes the luminol with the concomitant production of light, the intensity of which increases in the presence of a chemical intensifier (phenol). The light was then detected by exposing the blot to a photographic plate in a dark room for 30 s. Mouse monoclonal anti-tubulin antibody (1:4000) was used as a control in a separate blot, detected by peroxidase-conjugated goat anti-mouse Ig. Two groups of bands were selected on the basis of their higher/lower density and protein content: a group containing bands number 1, 2, 7, 8, and 9, and a group containing number 3, 4, 5, 6, and 10. They were compared for statistical analysis.

To confirm the morphological data obtained by comparing the expression of Cu, Zn-SOD in the epidermis, random samples of tissue fixed in formalin for 24 h and embedded in paraffin were sectioned at a thickness of 10 μm. With the aid of a stereoscopic microscope, a manual microdissection was performed eliminating the epidermis with a scalpel from some sections. The dermis and the entire skin section were then transferred into test tubes in order to evaluate the presence of Cu, Zn-SOD in the epithelial component only. The sections contained in the tubes were dewaxed, by adding 500 μl of iso-octane and vigorously shaken by vortexing for 10 s; after which 37.5 μl of methanol were added, followed by further shaking. After centrifugation at 15,000 g at 4°C for 20 min, the supernatant was removed and the pellet was left to dry for 5 min. The pellets were then heated in 30 μl of lysis buffer (2% SDS, 10% Glycerol, 0.05M DTT, 1mM EDTA, 20mM pH 7.2Tris-HCl) for 20 min. at 100°C and then incubated for 2 h at 60°C. Pre-heating at 100°C guarantees antigenic recovery, unmasking the protein cross-links caused by formalin fixation (10). The extracts were then frozen at −80°C until the time of protein quantitation.

## Results

### Immunohistochemistry

#### Epidermis

**Figure 1:**
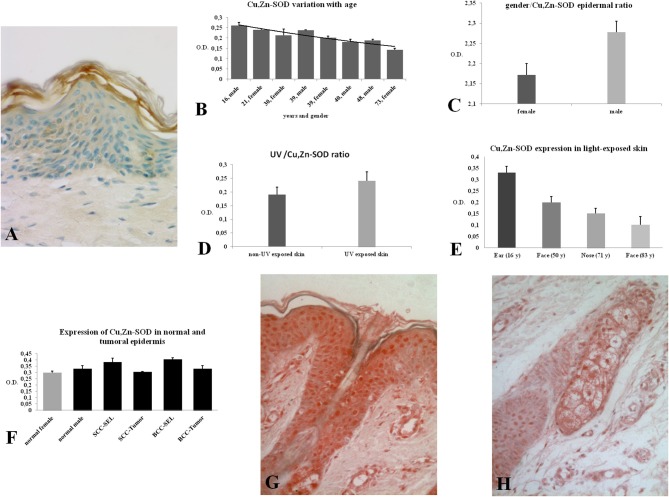
**(A)** Immunoreactivity of Cu, Zn-SOD in human skin. Cu, Zn-SOD immunoreactive cells are evident throughout the epidermis but also in the dermis. Development with peroxidase. 25x magnification. **(B)** Expression of Cu, Zn-SOD with reference to age in human epidermis. CU, Zn-SOD densitometric values are consistently higher in males than females during aging. The trend indicates a decreasing value of Cu, Zn-SOD expression with age, independent of gender, and female values are always positioned under the trend line. Of course this and following graphs do not express an absolute value. **(C)** Expression of Cu, Zn-SOD with reference to gender in human epidermis. As shown in **(B)**, CU, Zn-SOD densitometric evaluation in human epidermis shows a significantly higher level in males than females. *p* < 0.05. **(D)** Expression of Cu, Zn-SOD with reference to UV exposure in human epidermis. Densitometric Cu, Zn-SOD analysis of UV exposed vs. UV non exposed regions. UV exposed skin contains significantly more immunoreactive Cu, Zn-SOD than non-UV exposed skin. *p* < 0.05. **(E)** Expression of epidermal Cu, Zn-SOD in UV-exposed head regions. Ear, face and nose of UV-exposed human skin with increasing age were compared. OD levels decrease proportionally to age. **(F)** Cu, Zn-SOD expression in normal and tumoral human epidermis. Superior epithelial layers (SEL) of male basal cell carcinoma (BCC-SEL)-and of male spinocellular carcinoma (SCC-SEL) show higher densitometric values than deeper basal and squamous tumors. **(G)** Expression of Cu, Zn-SOD in a hair follicle. Cu, Zn-SOD immunopositivity is shown as a red stain. Note that nuclei are also immunoreactive. Development with alkaline phosphatase. 25x Magnification. **(H)** Expression of Cu, Zn-SOD in a sebaceous gland. Immunoreactivity of Cu, Zn-SOD in sebaceous glands is localized to the cytoplasm and nuclei of the clear secreting cells. Development with alkaline phosphatase. 25x Magnification.

In this study the skin and its annexes in the human integument system were examined. The expression of Cu, Zn-SOD in the human epidermis was analyzed in different skin areas from healthy subjects of both sexes and of different ages. Immunoreactivity for Cu, Zn-SOD was detected in the various layers of the epidermis (Figure 1A) and in the different cell types. Cytoplasmic distribution of Cu, Zn-SOD-immunoreactivity was comparable with immunoreactivity to cytokeratin but densitometric measurements carried out through the various layers of the epidermis showed variable SOD distribution. We did not measure cytokeratin, but would expect variations because of the different density of tonofilaments throughout the epidermal layers.

The skin areas analyzed were from the ear, back, breast, scalp, arm, thigh, and face. Some samples such as the ear and the face are strongly immunoreactive, others, such as the arm, show lower immunoreactivity. In the remaining samples no significant differences were noted (Table 1A). Generally, the horny layer expresses more Cu, Zn-SOD immunoreactivity than basal layer. This experiment was gender-blind. The results confirmed the higher SOD level in males. The densitometric values in the various epidermal layers of different subjects were then compared (Table 1B). At the level of the horny layer Cu, Zn-SOD-immunoreactivity in young women (<40) tends to be slightly lower than in older women (>40). However, despite the slight differences in horny and basal layer, the total value (horny + basal) is still higher in younger than in older females. In males, average densitometric values in the epidermis are slightly higher. At the level of the basal layer in all the samples examined there is generally a lower Cu, Zn-SOD immunoreactivity than in the horny layer. In women in general and in men the ear and the face among the examined samples are the regions richer in Cu, Zn-SOD than the arm (Tables 1B,C).

**Table 1A:** Presence of Cu, Zn-SOD within several epidermal areas.

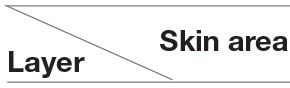	**Breast (*n* = 3)**	**Scalp (*n* = 3)**	**Ear (*n* = 3)**	**Arm (*n* = 3)**	**Thigh (*n* = 3)**	**Face (*n* = 5)**	**Back (*n* = 4)**
Horny	0.25 ± 0.06	0.24 ± 0.039	0.528 ± 0.13	0.17 ± 0.03	0.34 ± 0.034	0.29 ± 0.063	0.26 ± 0.048
Basal	0.22 ± 0.07	0.217 ± 0.056	0.448 ± 0.16	0.166 ± 0.04	0.19 ± 0.062	0.26 ± 0.06	0.21 ± 0.053
Total	0.235 ± 0.06	0.228 ± 0.047	0.488 ± 0.14	0.168 ± 0.035	0.265 ± 0.03	0.275 ± 0.06	0.235 ± 0.05

*Mean densitometric values of Cu, Zn-SOD-immunoreactivity in the basal and horny layers of various areas of human epidermis. Generally, the horny layer expresses more Cu, Zn-SOD immunoreactivity than the basal layer. For this experiment random genders were used that confirmed the results illustrated in Figure 1F. The number beside the skin area represents the number of samples analyzed*.

**Table 1B:** UV exposure, gender, health status and women age.

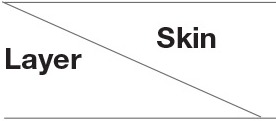	**UV exposed** **(*n* = 3)**	**Non exposed** **(*n* = 3)**	**Man** **(*n* = 3)**	**Woman** **(*n* = 3)**	**Healthy** **(*n* = 3)**	**Sick (e)** **(*n* = 3)**	**Women <40** **(*n* = 3)**	**Women > 40** **(*n* = 3)**
Horny	0.33 ± 0.072	0.23 ± 0.048	0.3 ± 0.06	0.274 ± 0.06	0.296 ± 0.06	0.23 ± 0.052	0.27 ± 0.059	0.278 ± 0.06
Basal	0.29 ± 0.083	0.226 ± 0.055	0.26 ± 0.07	0.258 ± 0.05	0.249 ± 0.07	0.22 ± 0.05	0.28 ± 0.075	0.236 ± 0.04
Total	0.31 ± 0.077	0.228 ± 0.05	0.28 ± 0.06	0.266 ± 0.055	0.27 ± 0.066	0.2 ± 0.051	0.275 ± 0.067	0.257 ± 0.05

*Mean densitometric values of Cu, Zn SOD-immunoreactivity in the epidermal horny and basal layers. These data show that UV exposed vs. UV non-exposed skin, male vs. female skin, young vs. old people's skin, and healthy vs. sick skin express SOD enzyme in the horny layer generally at higher level compared with basal layer. (e, epithelioma)*.

**Table 1C:** Dermis vs. epidermis Cu, Zn SOD-expression.

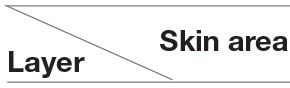	**Breast (*n* = 3)**	**Back (*n* = 4)**	**Scalp (*n* = 3)**	**Ear (*n* = 3)**	**Arm (*n* = 3)**	**Leg (*n* = 3)**	**Face (*n* = 5)**
Epidermis	0.235 ± 0.06	0.235 ± 0.05	0.228 ± 0.047	0.488 ± 0.10	0.168 ± 0.03	0.265 ± 0.03	0.275 ± 0.06
Papillary dermis	0.139 ± 0.022	0.126 ± 0.023	0.108 ± 0.03	0.239 ± 0.073	0.081 ± 0.03	0.1 ± 0.029	0.15 ± 0.027
Reticular dermis	0.136 ± 0.029	0.115 ± 0.031	0.095 ± 0.038	0.21 ± 0.066	0.094 ± 0.03	0.09 ± 0.021	0.159 ± 0.031
Dermis: total	0.138 ± 0.02	0.12 ± 0.027	0.1 ± 0.034	0.224 ± 0.06	0.087 ± 0.03	0.095 ± 0.02	0.154 ± 0.029
Dermis/epidermis ratio	0.186 ± 0.04	0.17 ± 0.038	0.16 ± 0.04	0.356 ± 0.08	0.127 ± 0.03	0.18 ± 0.025	0.21 ± 0.03

*The mean densitometric values in the papillary and reticular layers of the dermis in various, blindly selected areas. These data confirm that epidermal Cu, Zn-SOD expression is higher than in the dermis*.

The expression of Cu, Zn-SOD is higher in young subjects and tends to decrease gradually with age (Figure 1B) of both sexes. Densitometric analysis detects a slight difference in epidermal expression of the enzyme in healthy subjects of different gender, showing higher levels in males than in females (Figure 1C). Immunoreactivity of Cu, Zn-SOD is greater in UV-exposed skin areas than non-UV-exposed areas (Figure 1D). In UV-exposed regions the immunoreactivity of the enzyme is influenced by age; the highest value is in the subject of 16 years and the lowest in the subject of 83 years (Figure 1E).

Finally, the expression of Cu, Zn-SOD in the epidermis of healthy subjects and of patients with basal cell and spinocellular carcinomas was compared.

The expression of immunoreactive SOD in the epidermis of subjects with basal cell or spinocellular carcinoma was greater than that of normal subjects. However, in the tumor cells invading the dermis, expression was lower than in normal subjects (Figure 1F).

#### Dermis

Immunohistochemical analysis of the Cu, Zn-SOD of the dermis of human skin shows mild immunoreactivity compared with that of the epidermis in all the areas examined including the tunica intima of the blood vessels. Densitometric analysis of its SOD-immunoreactivity confirms the morphological observations. Both the papillary and reticularis zones of the dermis area were analyzed. The distribution in the various subjects examined is shown in Table 1D.

**Table 1D:** Hair follicle.

**Skin area**	**Leg (*n* = 3)**	**Nose (*n* = 3)**	**Ear (*n* = 3)**
Hair follicle	0.21 ± 0.087	0.398 ± 0.1	0.54 ± 0.21

*Mean densitometric values of Cu, Zn-SOD immunoreactivity in the hair follicle of nose and ear areas exceed the leg values*.

#### Hair Follicle

The hair follicle, the bulb and the papilla were analyzed. The papilla, like the dermis, shows poor Cu, Zn-SOD-immunoreactivity except for blood vessels. The cells of the external epithelial sheath of the hair bulb, which are continuous with the epidermis, are intensely immunoreactive (Figure 1G), unlike the cells of the internal epithelial sheath. The cells of the cuticle facing the inner epithelial sheath appear intensely immunoreactive, while the cells of the cuticle facing the cortical area of the bulb are non-immunoreactive to anti-Cu, Zn-SOD. The central area comprising the bulbar cortical and medulla shows SOD-immunoreactive stellate cells. Above the hair bulb, there are positive cells in the outermost layer that corresponds to the invaginated part of the surface epidermis. The erector pili muscle, where visible, is immunoreactive. Occasional densitometric measurements were carried out at the hair follicle outer sheath.

#### Sweat and Sebaceous Glands

Apocrine sweat glands and sebaceous glands were examined. In both types of gland the intensity of the immunoreaction was quite high and variations between the samples were negligible, except that the glands of the most exposed areas of skin seemed to contain greater amounts of Cu, Zn-SOD (Figure 1H, see Figures S1, S2). Densitometric measurements confirmed the morphological observations (Table 1E).

**Table 1E:** Sweat and sebaceous glands.

**Skin**	**Nose (*n* = 3)**	**Ear (*n* = 3)**	**Scalp (*n* = 3)**	**Face (*n* = 3)**	**Arm (*n* = 3)**	**Breast (*n* = 3)**
Apocrine sweat gland	0.56 ± 0.22	_	0.157 ± 0.088	0.24 ± 0.090	0.27 ± 0.1	0.29 ± 0.1
Sebaceous gland	0.289 ± 0.086	0.45 ± 0.2	_	0.21 ± 0.05	_	_

*Mean densitometric values of CuZn-SOD-immunoreactivity in nose sweat and sebaceous glands exceed the values in the other areas considered*.

### Western Blot

**Figure 2:**
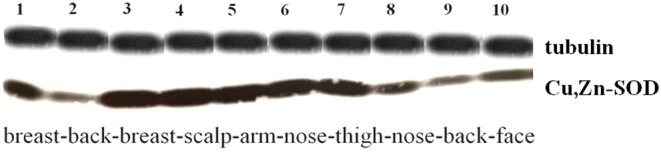
Western Blot analysis on nitrocellulose membranes incubated with anti-Cu, Zn-SOD polyclonal antibody (above: tubulin 56 kD, below: Cu, Zn-SOD 14 kD). Specimens were randomly selected and blotted in the following order: #1. Breast, lower inner quadrant; #2. Back, supragluteal skin; #3. Breast, upper outer quadrant; #4. Head, scalp; #5. Arm, forearm skin; #6. Head, nose; #7. Thigh, rectus femoris skin; #8. Head, nose; #9. Back, scapular skin; #10. Head, face (tumor). Western Blot analysis mostly confirms the histo-densitometric results. Specimens # 1, 2, 7, 8, 9, and 10 were grouped and the *T*-test was compared with that of the exposed group (specimens #3, 4, 5, 6). *p* < 0.05.

The presence of Cu, Zn-SOD in the epidermis was confirmed by the molecular study. Western Blot analysis on skin fresh tissue detects immunoreactive bands for SOD with a MW of 21 KDa. The densitometric quantification of Cu, Zn-SOD in the various tissues used, not shown here, indicates a variability of the enzyme content in the various cutaneous areas studied (Figure 2). The MW with which the membrane normalization was carried out is approximately 56 KDa.

The two groups of differently selected bands were compared by performing a two-tailed heteroscedastic t-student analysis and appeared to be significantly different. The only #5 specimen, arm skin, that we expected to belong to the non-exposed group, didn't match the other components of the group.

Western Blot analysis of the skin slides reveals different SOD-immunoreactive bands in the whole skin and in the dermis alone (Table 1F).This experiment tells us that dermis produces its own Cu, Zn-SOD.

**Table 1F:** Skin western blot.

**Sample**	**Standard**	**Dermis**	**Skin**
Quantity (ng SOD1/mg of sample)	100	11	18

*Western Blot analysis with anti-SOD1 polyclonal antibody on ffpe sections*.

## Discussion

During the mitochondrial respiration process, used by cells for energy production, small amounts of partially reactive oxygen species are generated. These are free radicals, responsible for modifications in the amino acids of proteins, which frequently induce functional or structural changes of enzymatic proteins. Defense against damage by these reactive oxygen species in the skin is regulated by various elements among which Cu, Zn-SOD appears to be the main antioxidant enzyme. Our study aims to evaluate the expression of Cu, Zn-SOD in the human epidermis, since current knowledge about the presence of this enzyme is quite limited and mainly comes from studies on non-human mammalian skin. In addition, most of the studies were performed on whole skin, not separating the epidermis from the dermis. In this study, to avoid these drawbacks, morphometric measurements were performed by means of densitometry on the epidermis alone and a western blot analysis was performed on separate samples of whole skin and dermis only. Preliminary data on protein extraction by manual microdissection of epidermis directly from histological sections confirm that protein expression values are different in the epidermis and in the dermis. The epidermis is certainly more exposed to external agents than the dermis, so the production of ROS and the defensive system against them are thought to be different (7). However, most of these studies were biochemical and carried out on animal cell cultures, which do not allow precise evaluation of the enzyme localization. WB experiments on samples of whole fresh skin confirmed the histodensitometric results with the exception of the forearm (#5). Specimen had been selected ignoring their original anatomical region. Therefore, given its high density value of specimen #5, we expected that was an UV-exposed type of skin. Unexpectedly, it corresponded to forearm. Examination of the case history showed that the patient was female and the operation was done in summer time. Therefore, it is likely that the forearm had been exposed to UV which could explain the anomalous result.

To evaluate the immunoreactivity of Cu, Zn-SOD in the human epidermis, immunohistochemical methods were applied, using a monoclonal antibody to Cu, Zn-SOD, and subsequent densitometric analysis, to quantify the intensity of the signal linked to DAB on histological sections. We show that enzyme immunoreactivity varies according to age, with higher levels in young than in old subjects. These results agree with the data in the literature, according to which the enzymatic activity of Cu, Zn-SOD tends to decrease with age, particularly after 60 years (11). From this it can be deduced that with advancing age and reduction in the activity of antioxidant enzymes, there is an accumulation of ROS and therefore an increase in oxidative stress.

Our study on the epidermis of skin areas exposed and not exposed to light, shows that the presence of Cu, Zn-SOD is greatest in regions exposed to UV rays, like the face. These data are in contrast with those presented by Hellemans (2), according to which there is no seasonal variation in the concentration of Cu, Zn-SOD in either exposed or unexposed skin. The discrepancy in our findings is probably because there are no studies specifically conducted on the epithelial component only of human skin; assuming that, in addition to oxidative metabolism and inflammation, the most important factor in the production of ROS is exposure to UV rays. Since Cu, Zn-SOD increases in response to an accumulation of ROS this increase would confirm the protective role of the enzyme against radiation-induced skin damage. In fact, some studies show that the pre-treatment of skin with topical SOD protects the skin from inflammatory reactions induced by UVA rays (12).

We also found that enzyme immunoreactivity, although increased in the UV-exposed regions, is always influenced by age with high expression in the younger subjects and much lower in the older. Furthermore, the enzyme immunoreactivity was higher in males than females; this has not been previously reported.

According to the literature, the presence of Cu, Zn-SOD tends to decrease in carcinomas and epitheliomas of the skin (7, 13). We confirm this finding, although the reason for the decrease remains obscure. We also compared the expression of Cu, Zn-SOD in the epidermis of healthy subjects with that in patients with basal cell and spinocellular carcinomas, considering, in the tumors, both the surface epithelium and the cells that extend and proliferate in the dermis. What emerges from our study is that patients with carcinoma have higher values of enzyme expression in the superficial epidermis than in tumor cells located in the dermis. The low level of Cu-Zn SOD in the tumor might be due to inhibition by the hydrogen peroxide originally produced by the enzyme's action on ROS. This could prevent the further production of oxygen and hydrogen peroxide from ROS which would inhibit the enzymes involved in the subsequent reactions (14, 15) and thus contributes to the hypoxic conditions favorable for tumor growth. Experimental evidence is needed to confirm this speculation. The literature on this topic is limited and inconsistent. Although most of the studies detect a reduction of Cu, Zn-SOD in carcinomas (7, 16), there are exceptions. For example, in colorectal cancer there are no differences between tumor tissue and normal tissue, while some studies on lung cancer show higher levels of Cu, Zn-SOD compared to normal samples (17). Other studies show that antioxidant defenses, although highly efficient, have a limited capacity and may decrease, causing an increase in ROS in the skin (18). In fact at low ROS levels there was an increase of anti-oxidants, as an adaptive response, but at higher levels the enzymes lost their capacity and their expression was reduced. Furthermore, it was shown that a single exposure to UV rays in human skin involved a transient reduction of Cu, Zn-SOD activity; however, after chronic UVB irradiation, Cu, Zn-SOD epidermal activity was induced (6).

In conclusion, our study contributes to knowledge concerning skin Cu, Zn-SOD and confirms its protective role against damage induced by ROS. Until more is known about the role of this important family of enzymes in skin cancer, the current topical application of SOD-containing cream as a preventative seems to be the best approach. The enzyme first increases to counteract the accumulation of superoxide anions, but when the damage has become chronic, it is probably inhibited by the product of the catalyzed reaction, hydrogen peroxide, and is reduced, thus favoring the action of ROS and other reactive species.

## Data Availability Statement

All datasets generated for this study are included in the article/Supplementary Material.

## Ethics Statement

Ethical review and approval was not required for the study on human participants in accordance with the local legislation and institutional requirements. Written informed consent for participation was not required for this study in accordance with the national legislation and the institutional requirements.

## Author Contributions

VC designed and analyzed experiments and wrote the manuscript. GA conducted the experiments, interpreted, and analyzed data. SV advised on the manuscript and its content. All authors participated in discussion of results, manuscript editing, and approved the final manuscript.

### Conflict of Interest

The authors declare that the research was conducted in the absence of any commercial or financial relationships that could be construed as a potential conflict of interest.
