# Supplementary material for: Copper/Zinc-Superoxide Dismutase in Human Epidermis: An Immunochemical Study
Source: Front Med (Lausanne). 2019 Nov 13;6:258. doi: 10.3389/fmed.2019.00258 (PMC6874168; doi:10.3389/fmed.2019.00258)
Supplement: Supplementary file 1 [file Table_1.doc]

**Table 1Suppl** **Antibodies used with immunochemical methods**. Rabbit (rb) anti-ACTH and Mouse (ms) anti-insulin antibodies were used as positive control for immunocytochemistry as well as monoclonal anti α-tubulin was used for W.B.

| **ANTIBODY** | **DILUTION** | **COMPANY** | **CAT. NUMBER** |
| --- | --- | --- | --- |
| ms monoclonal anti-SOD | 1/75 | Sigma St.Louis, MO, USA | S2147 |
| goat (gt)anti-ms Ig conjugated with peroxidase | 1/100 | Dako A/S, Denmark | P0447 |
| gt anti-ms Ig conjugated with alkaline phosphatase | 1/50 | Dako A/S, Denmark | D0486 |
| **m**s monoclonal anti-insulin | 1/4000 | Sigma, St.Louis, MO, USA | I-2018 |
| **rb** anti-ACTH | 1/4000 | Dako A/S, Denmark | A571 |
| monoclonal mouse anti-Human cytokeratin (ctk) | 1/50 | Dako A/S, Denmark | M821 |
| **rb** anti-SOD1 | 1/1000 | Santa Cruz Biotechnology (USA) | sc-11407 |
| Monoclonal anti-α-Tubulin | 1/4000 | Sigma, St.Louis, MO, USA | T5168 |
