# Supplementary figures and images for: Copper/Zinc-Superoxide Dismutase in Human Epidermis: An Immunochemical Study
Source: Front Med (Lausanne). 2019 Nov 13;6:258. doi: 10.3389/fmed.2019.00258 (PMC6874168; doi:10.3389/fmed.2019.00258)

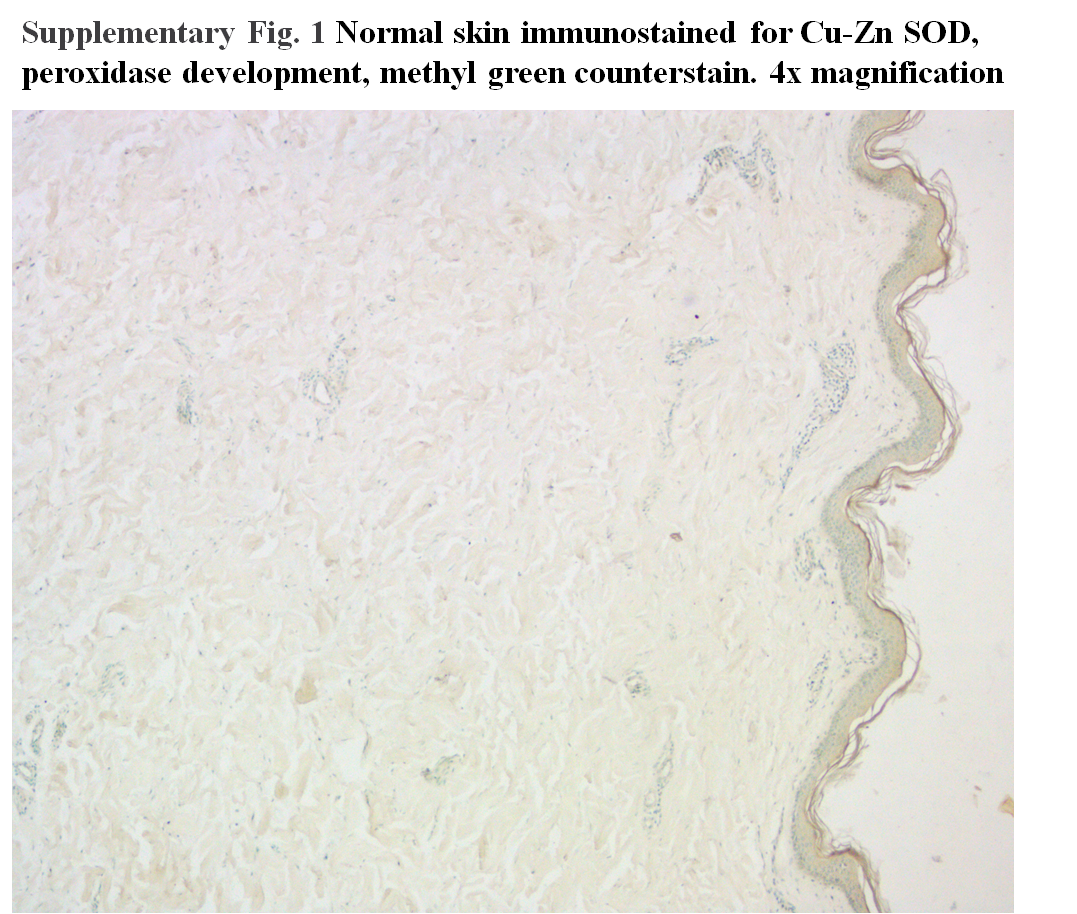

Supplement: Supplementary file 2 [file Image_1.TIF]

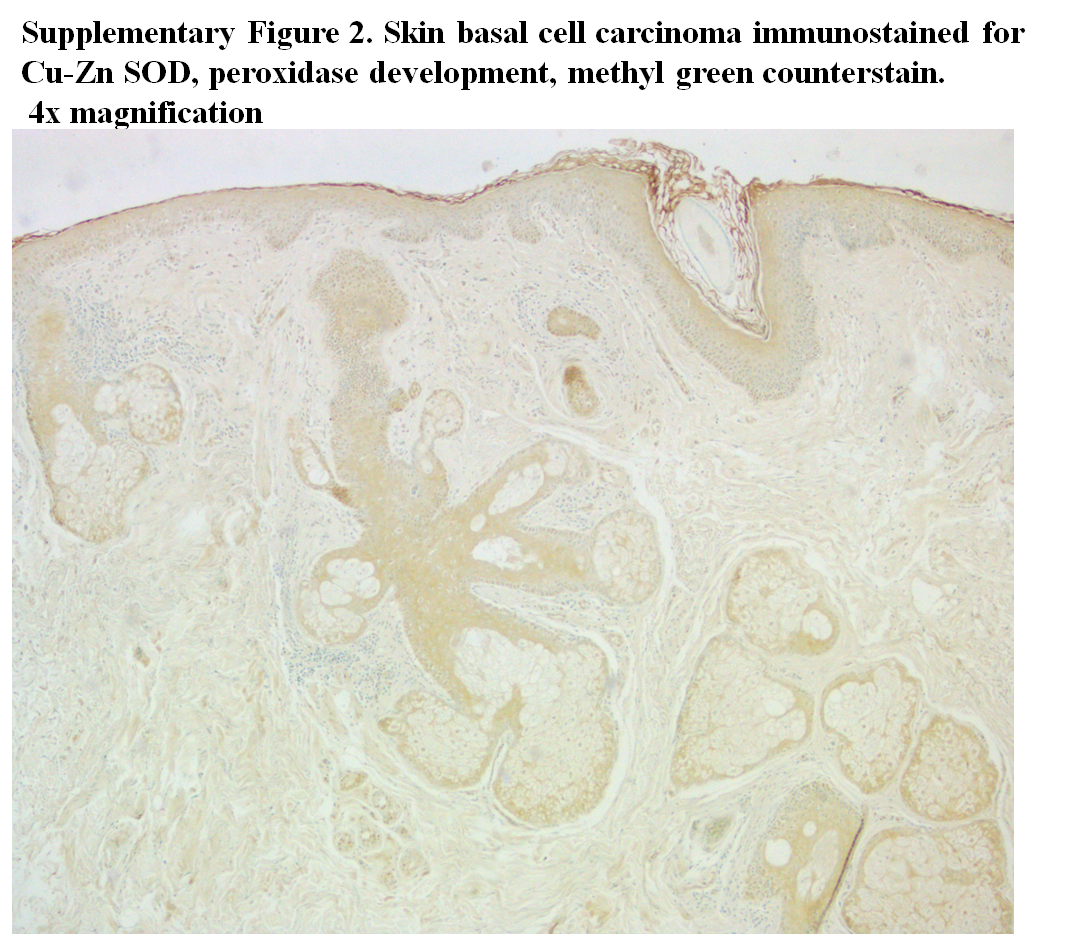

Supplement: Supplementary file 3 [file Image_2.TIF]
